# Proteomic profiling identifies SPP1 associated with rapidly progressive interstitial lung disease in anti-MDA5-positive dermatomyositis

**DOI:** 10.1186/s13075-023-03243-z

**Published:** 2024-01-02

**Authors:** Yulu Qiu, Xiaoke Feng, Chang Liu, Yumeng Shi, Lingxiao Xu, Hanxiao You, Lei Wang, Chengyin Lv, Fang Wang, Wenfeng Tan

**Affiliations:** 1 Department of Rheumatology, The First Affiliated Hospital of Nanjing Medical University, 300 Guangzhou Road, Nanjing, 210029 Jiangsu Province China; 2 Department of Traditional Chinese Medicine, The First Affiliated Hospital of Nanjing Medical University, Nanjing, Jiangsu China; 3 Integrated Traditional Chinese and Western Medicine Institute of Nanjing Medical University, Nanjing, Jiangsu China; 4 Department of Cardiology, The First Affiliated Hospital of Nanjing Medical University, 300 Guangzhou Road, Nanjing, 210029 Jiangsu Province China

**Keywords:** Dermatomyositis, MDA5, Interstitial lung disease, RP-ILD, Biomarker

## Abstract

**Background:**

Anti-melanoma differentiation-associated gene five antibody positive (MDA5^+^) dermatomyositis (DM) is significantly associated with rapidly progressive interstitial lung disease (RP-ILD). Early detection of RP-ILD remains a major challenge. This study aims to identify and validate prognostic factors for RP-ILD in MDA5^+^ DM patients.

**Methods:**

Plasma samples from 20 MDA5^+^ DM patients and 10 healthy controls (HC) were collected for proteomic analysis using liquid chromatography-tandem mass spectrometry (LC–MS/MS) analysis. The proteins of interest were validated in independent samples (20 HC, 20 MDA5^+^ DM with RP-ILD, and 20 non-RP-ILD patients) with enzyme-linked immunosorbent assay** (**ELISA).

**Results:**

A total of 413 differentially expressed proteins (DEPs) were detected between the MDA5^+^ DM patients and HC. When comparing DEPs between RP-ILD and non-RP-ILD patients, 79 proteins were changed in RP-ILD patients, implicating acute inflammatory response, coagulation, and complement cascades. Six candidate biomarkers were confirmed with ELISA. Secreted phosphoprotein 1 (SPP1), serum amyloid A1 (SAA1), and Kininogen 1 (KNG1) concentrations were significantly elevated in RP-ILD patients than those in non-RP-ILD patients and HC. In the different clinical subgroups, SPP1 was particularly elevated in the high-risk RP-ILD subgroup of MDA5^+^ DM.

**Conclusion:**

This study provides novel insights into the pathogenesis of RP-ILD development in MDA5^+^ DM and suggests the plasma protein SPP1 could serve as a potential blood biomarker for RP-ILD early warning.

**Supplementary Information:**

The online version contains supplementary material available at 10.1186/s13075-023-03243-z.

## Background

Anti-MDA5 antibody-positive dermatomyositis (MDA5^+^ DM), a distinct subtype of idiopathic inflammatory myopathies (IIM), is significantly associated with interstitial lung disease (ILD), particularly the rapidly progressive interstitial lung disease (RP-ILD). MDA5^+^ DM with RP-ILD is often resistant to traditional treatment, and despite aggressive interventions, the mortality rate is still as high as 50-70% [1, 2]. Hence, early identification of high-risk RP-ILD patients is a vital aspect of improving prognosis.

MDA5^+^ DM is a heterogeneous disease, with substantial variability in clinical presentation, treatment response, and patient outcomes. About 30 ~ 50% of patients exhibited the life-threatening RP-ILD [1, 3], conversely, while about 60% of patients MDA5^+^ DM displayed mild symptoms with good prognosis. Therefore, accurate prediction of patients at high risk of RP-ILD may aid in decision-making on therapeutic strategies and assist in preventing overtreatment of “low-risk” patients.

During the last decade, efforts have been made to identify prognostic markers in MDA5^+^ DM patients. Male, old age, short disease duration, skin ulceration, and forced vital capacity are suggested as risk factors for RP-ILD [2, 4]. Some serum markers like the co-existence of anti-MDA5 antibody and anti-RO-52 antibody, elevated serum C-reactive protein (CRP), ferritin level, and Krebs von den Lungen-6 (KL-6) levels have been linked to poor outcome in RP-ILD patients [1, 2, 5]. However, due to the exact mechanisms underlying RP-ILD development have not yet been determined, most prognostic risk research mainly focused on the epidemiological and clinical characteristics of MDA5^+^ DM. Currently, no reliable biomarkers for predicting RP-ILD or morality in MDA5^+^ DM are available.

We hypothesized that although the specific pathogenesis and mechanism of RP-ILD are largely unknown, the rapidly progressive lung injury can induce distinctive molecular changes. Proteins with these changes can be detected in the peripheral blood of MDA5^+^ DM patients, potentially serving as surrogate markers for disease progression. High-throughput proteomics offers a promising approach to identify these biomarkers for RP-ILD. In the current study, we performed proteomic analysis to explore potential pathways and biomarkers that linked to RP-ILD development in MDA5^+^ DM patients.

## Methods

### Study populations

A total of 40 MDA5^+^ DM patients were recruited between October 2021 and October 2022 at the First Affiliated Hospital of Nanjing Medical University. All patients have met the Bohan and Peter and Sontheimer’s criteria [6]. This study adheres to the Declaration of Helsinki and was approved by the Ethics Committee of the First Affiliated Hospital of Nanjing Medical University (ID: 2020-SR-265), and all participants provided written informed consent. Anti-MDA5 antibodies were tested by immunoblot testing (Euroimmun, Lubeck, Germany). ILD or RP-ILD was defined as previously described [2]. Patients with other concurrent autoimmune diseases were excluded. Some chronic comorbidities were recorded, including arthritis, diabetes, kidney disease, and cardiovascular disease. The definitions of these chronic diseases were as previously reported [7–10].

### Sample collection

Blood samples were collected in EDTA-treated vacuum tubes and centrifugated a 3000 rpm for 10 min at room temperature. After the centrifugation, plasma was stored frozen at − 80 °C for LC–MS/MS or ELISA analysis.

### LC–MS/MS analysis

Eight microliters of each plasma sample were processed with the High-Select™ Top14 Abundant Protein Depletion Resin according to the manufacturer’s instruction (A36372, Thermo). Following depletion, proteins were subjected to LC–MS/MS with the Easy-nLC 1200 system coupled with a Q Exactive HF-X hybrid Quadrupole-Orbitrap (both Thermo Fisher Scientific, San Jose, USA) [11]. Purified peptides were separated on 25-cm HPLC columns with an inner diameter of 75 μm packed in-house with ReproSil-Pur C18-AQ 1.9 μm resin (Dr. Maisch GmbH). And about 1-μg peptides were loaded for each LC–MS/MS analysis. Raw data were acquired with a Data-Independent Acquisition method and analyzed by Spectronaut 15.0 (Biognosys AG, Switzerland). *Q* value (FDR) cutoff was set to 1% at the peptide and protein level. The average top 3 filtered peptides which passed the 1% *Q* value cutoff were used to calculate the major group quantities.

### Principal component analysis (PCA) analysis

“FactoMineR [12]” and “factoextra [13]” packages in R were utilized to conduct PCA. The “FactoMineR” package allowed us to derive key statistical information and perform the actual PCA calculation. The “factoextra” package contributed to the generation of insightful graphical representations to interpret the results of the PCA effectively.

### Differential protein expression analysis and visualization

Statistical analysis of original data was conducted using the Wilcoxon test and the Kruskal–Wallis test. By applying a significance threshold of *p* < 0.05, we identified proteins that displayed differential expression among the groups. To depict the differences in differentially expressed proteins (DEPs) above, we utilized volcano plots to examine the distribution and selected the top 50 DEPs for visualization in heatmaps.

The utilization of a volcano plot, a type of scatterplot, has been employed as a means of visualizing the dissimilarities in gene expression between two distinct groups [14]. The *x*-axis represents the log2 fold change, while the *y*-axis depicts the -log10 (*p*-value). Notably, upregulated genes are depicted as red dots, downregulated genes as blue dots, and genes that lack statistical significance are depicted as black dots. The title positioned above the diagram denotes the specific comparison groups.

Heatmaps are another visualization tool that represents differential expression data in a grid-like fashion [15]. Each cell indicates the gene expression level of a particular sample and the color indicates the gene’s expression. In our work, high-expression proteins are depicted as red squares and low-expression proteins as blue squares.

The production of the heatmap and volcano plot was carried out by using “pheatmap” [16] and “ggpubr” [17] packages, respectively, in R software.

### Function enrichment analysis

An online platform of Metascape was used for gene annotation and analysis, revealing biological significance and mechanisms of gene sets [18]. The gene set of interest was uploaded to the website according to the developers’ requirements, and a large number of enrichment entries from multiple databases (GO Biological Processes, KEGG Pathway, WikiPathways, etc.) were obtained. The “ggpubr” package in R software was used to process the original file to a bar chart [17]. Its *y*-axis indicates entries with different enrichments, while the *x*-axis corresponds to -log (*P* value). The color of the bars indicates the number of genes in annotation pathways.

Chord diagram is another visual method that can display genes and their corresponding enrichment pathways. The enrichment pathway of interest and the corresponding genes were visualized in the form of a chord diagram using the “circlize” [19] package in the R software.

### Trend analysis

The trend analysis was carried out on the OmicShare tools, a free online platform for data analysis (https://www.omicshare.com/tools). The analysis was based on Short Time-series Expression Miner (STEM) to cluster samples at different stages and analyze their expression patterns [20]. Unlike screening different genes according to a specific threshold, we pay more attention to the specific changes of proteins at multiple times. All operations are based on the system default parameters, and the maximum output profile number is 8 [21].

### Enzyme-linked immunosorbent assay (ELISA)

Following the manufacturer’s instructions, commercial ELISA kits were utilized for quantifying the levels of serum amyloid A1 (SAA1) (Boster Bio, China), V-Set And Immunoglobulin Domain Containing 4 (VSIG4), Coagulation Factor XI (F11) (both from CUSABIO, China), Coagulation Factor XIII A Chain (F13A1), Secreted phosphoprotein 1 (SPP1) and Kininogen 1 (KNG1) (all from Uscn life Science, China) in plasma samples from both patients and normal control subjects. The area under the receiver operating characteristic (ROC) curve (AUC) was used to evaluate the prediction performance of developing RP-ILD.

### Statistical analysis

Statistical analyses were performed using Prism 9 (GraphPad Software, La Jolla, USA), SPSS 20, and Rstudio, an integrated development environment for the R programming language (http://www.r-project.org). Data are presented as *n* (%) or median [interquartile range].

In mass spectrometry, the Wilcoxon rank sum test and Fisher’s exact test were used to compare continuous and categorical variables between the two patient sample groups for assessing statistical differences. Multi-group comparisons were conducted using a one-way ANOVA test. The generalized linear model (GLM) was employed to test the influence of potentially confounding variables, including chronic diseases such as arthritis, diabetes, renal disease, and cardiovascular disease on protein expression. The diagnostic performance of each protein was evaluated using the ROC curve, with SPSS calculating the area under the ROC curve, sensitivity, and specificity. The significance level was defined as *p* < 0.05.

## Results

### Patients’ characteristics

Demographics and baseline characteristics of participants are shown in Table 1. A total of 20 MDA5^+^ DM patients and 10 HC were included for proteomic analysis. No significant differences in age and sex were observed between groups. In the context of MDA5^+^ DM, 10 patients had RP-ILD, and 10 patients had non-RP-ILD. Compared with non-RP-ILD, patients with RP-ILD had higher all-cause mortality within 6 months and are more likely to have high levels of serum inflammatory markers, including erythrocyte sedimentation rate (ESR), CRP, and serum ferritin. The prevalence of chronic diseases (arthritis, diabetes, renal disease, and cardiovascular disease) showed no statistically significant difference between the two groups of patients.

**Table 1:** Clinical characteristics and laboratory features of the patients and healthy controls

Parameters	Healthy control (*n* = 10)	MDA5^+^non-RPILD (*n* = 10)	MDA5^+^RP-ILD (*n* = 10)	*p*-value
Female, *n* (%)	5 (50)	7 (70)	6 (60)	> 0.99
Patient age, years, median (interquartile range)	41.00 (38.00,58.00)	44.50 (31.25, 54.00)	45.50 (42.75, 54.50)	0.65
General condition/inflammation				
ALT, U/L, median (interquartile range)	-	40.50 (20.80, 55.40)	40.75 (25.50, 108.58)	0.55
AST, U/L, median (interquartile range)	-	54.20 (29.20, 68.90)	49.45 (35.38, 86.58)	0.55
LDH, U/L, median (interquartile range)	-	279.5 (243.00, 357.25)	401.00 (353.00, 424.00)	0.019
CK, U/L, median (interquartile range)	-	83.00 (42.75, 143.00)	87.50 (44.75, 111.75)	0.97
ESR, mm/h, median (interquartile range)	-	33.00 (23.00, 49.00)	40.00 (32.00, 54.00)	0.6
CRP, mg/L, median (interquartile range)	-	4.38 (3.05, 5.85)	9.76 (3.64, 20.05)	0.22
SF, ng/mL, median (interquartile range)	-	382.60 (177.70, 1,008.10)	799.80 (94.75, 1,854.75)	> 0.99
Immunosuppressive therapy, *n* (%)	-	0 (0%)	3 (30%)	0.21
Disease course, months, median (interquartile range)	-	3.00 (1.25, 4.00)	1.50 (1.00, 2.75)	0.38
Chronic disease status, *n* (%)				
Arthritis	0	3 (30)	2 (20)	0.63
Diabetes	0	0 (0)	1 (10)	> 0.99
Kidney disease	0	0 (0)	0 (0)	-
Cardiovascular disease	0	0 (0)	1 (10)	> 0.99
Skin and muscular manifestations, *n* (%)				
Gottron	-	7 (70)	8 (80)	> 0.99
Heliotrope	-	5 (50)	5 (50)	> 0.99
Mechanic hands	-	4 (40)	4 (40)	> 0.99
Muscle weakness	-	2 (20)	5 (50)	0.35
Myocardial involvement	-	1 (10)	0 (0)	0.63
Periungual erythema	-	3 (30)	5 (50)	0.65
Shawl signs	-	1 (10)	2 (20)	> 0.99
Skin erythema	-	6 (60)	3 (30)	0.37
Skin ulcers	-	0 (0)	1 (10)	> 0.99
V syndrome	-	2 (20)	3 (30)	> 0.99
Antibodies, *n* (%)				
MDA5				0.63
+	-	3 (30)	1 (10)	
+ +	-	3 (30)	3 (30)	
+ + +	-	4 (40)	6 (60)	
ANA	-	4 (40)	4 (40)	> 0.99
Ro52	-	4 (40)	10 (100)	0.011
Ro60	-	0 (0)	3 (30)	0.21
PL12	-	1 (10)	0 (0)	> 0.99
Ku	-	1 (10)	0 (0)	> 0.99

Data are presented as median (interquartile range) or case number (percentage)

*Abbreviations: RP-ILD* rapidly progressive interstitial lung disease, *ALT* alanine transaminase, *AST* aspartate transaminase, *LDH* lactate dehydrogenase, *CK* creatine kinase, *ESR* erythrocyte sedimentation rate, *CRP* C-reactive protein, *SF* serum ferritin, *MDA5* anti-melanoma differentiation-associated gene five, *ANA* antinuclear antibody

### Proteomic profiling of MDA5^+^ DM plasma

**Fig. 1:**
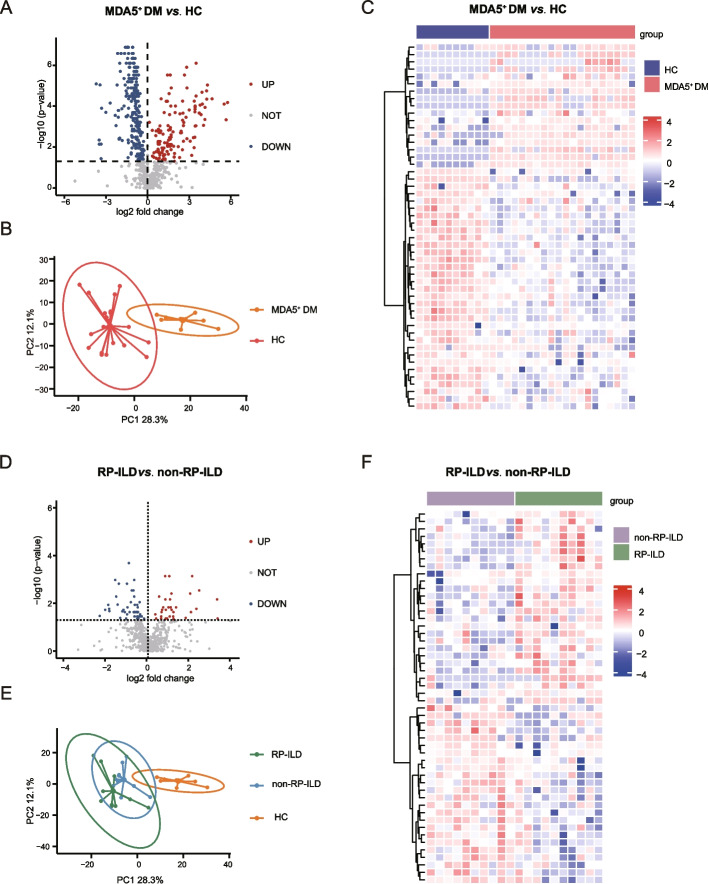
Differentially expressed proteins (DEPs) in MDA5^+^ DM and MDA5^+^ DM with RP-ILD samples. **A**, **D** Volcano plots depicted DEPs in comparison of MDA5^+^ DM versus HC, and MDA5^+^ DM with RP-ILD versus non-RP-ILD, respectively. The screening criteria were *p* < 0.05. **B**, **E** PCA cluster plots showed the overall distribution of HC and MDA5^+^ DM samples, and MDA5^+^ DM RP-ILD and non-RP-ILD samples. **C**, **F** Heatmaps showed the top 50 DEPs in comparisons of MDA5^+^ DM versus HC, and MDA5^+^ DM with RP-ILD versus non-RP-ILD, respectively. HC, healthy control

By LC–MS/MS analysis, we identified a total of 413 DEPs between the MDA5^+^ DM patients and HC (with criteria: *p* < 0.05) (Fig. 1A). PCA demonstrated a different distribution pattern between the two groups, based on the expression of protein in all samples (Fig. 1B). Of the DEPs, 159 proteins were upregulated, whereas 254 were significantly downregulated in the MDA5^+^ DM patients. The heatmap shows the top 50 DEPs (Fig. 1C) and the details of all proteins can be found in Additional file 1: Table S1.

### Proteomic profiling in MDA5^+^ DM with RP-ILD plasma

We next investigated the proteomic profile in the plasma of MDA5^+^ DM patients with RP-ILD. RP-ILD, non-RP-ILD patients, and HC could be clearly separated by PCA analysis in all samples (Fig. 1E). When comparing DEPs between RP-ILD and non-RP-ILD patients, we identified changes in a total of 79 proteins in RP-ILD patients. Among these proteins, 32 were upregulated (including FBLN2, SAA2, and SPP1) and 47 were down-regulated proteins (including DNM1L, FABP5, OIT3, ITGB3, etc.) in RP-ILD patients (Fig. 1D). The heatmap in Fig. 1F shows the top 50 of these proteins, and the details for all proteins can be found in Additional file 2: Table S2.

### Activated acute phase proteins, complement, and coagulation pathways in MDA5^+^ DM with RP-ILD plasma

**Fig. 2:**
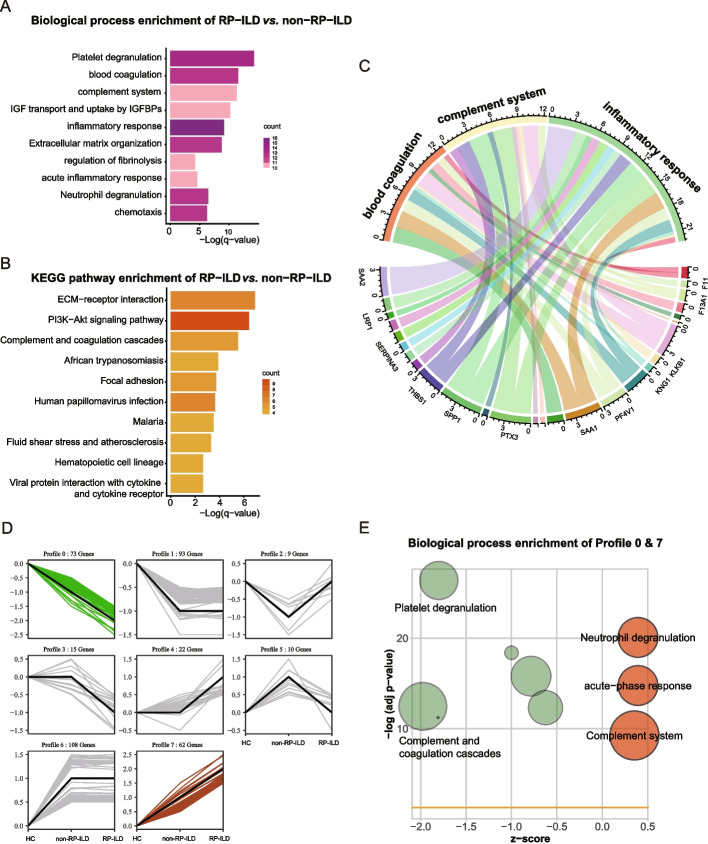
Proteomic features of MDA5^+^ DM with RP-ILD samples. The bar plots showed the most enriched biological process (**A**) and KEGG pathways (**B**) of DEPs. The screening criteria were *q* < 0.05. **C** The chord plot illustrated the interactions between the DEPs and the interested biological processes. **D** The line charts visualized the 8 STEM clusters based on protein expression in three stages of disease progression.** E** The bubble charts showed the most enriched biological process and KEGG pathways of proteins of profile 0 and profile 7

Pathway analysis and network enrichment analysis were used to explore the potential biological processes of DEPs (Fig. 2A and B). Five main biological processes were identified in RP-ILD patients: (1) platelet degranulation, (2) blood coagulation, (3) complement system, (4) IGF transport and uptake by IGFBPs, and (5) inflammatory response. And the relevant genes are exhibited in Fig. 2C. KEGG pathway enrichment analysis showed that the most significant enriched signal pathway was ECM-receptor interaction, PI3K-Akt signaling pathway, complement, and coagulation cascades. Several pathogenic infection pathways were identified using the KEGG pathway, including African trypanosomiasis, human papillomavirus infection, malaria, and viral protein interaction with cytokine and cytokine receptor.

STEM was used to analyze the protein expression pattern in HC, MDA5^+^ DM without RP-ILD, and MDA5^+^ DM with RP-ILD patients. A total of 8 patterns were identified (Fig. 2D). Profiles 0 and 7 represented gene sets that displayed continuous upregulation and downregulation, respectively. GO enrichment analysis of the proteins in profiles 0 and 7 characterizes the 5 most common GO terms into platelet degranulation, neutrophil degranulation, IGF transport and uptake by IGFBPs, hemostasis, and acute-phase response (Fig. 2E).

Taken together, our bioinformatics analysis revealed the molecular changes in the plasma of MDA5^+^ DM with RP-ILD patients, in comparison to the non-RP-ILD group. These changes imply involvement in acute inflammatory response, coagulation, and complement cascades.

### Validation of plasma proteins related to RP-ILD

**Fig. 3:**
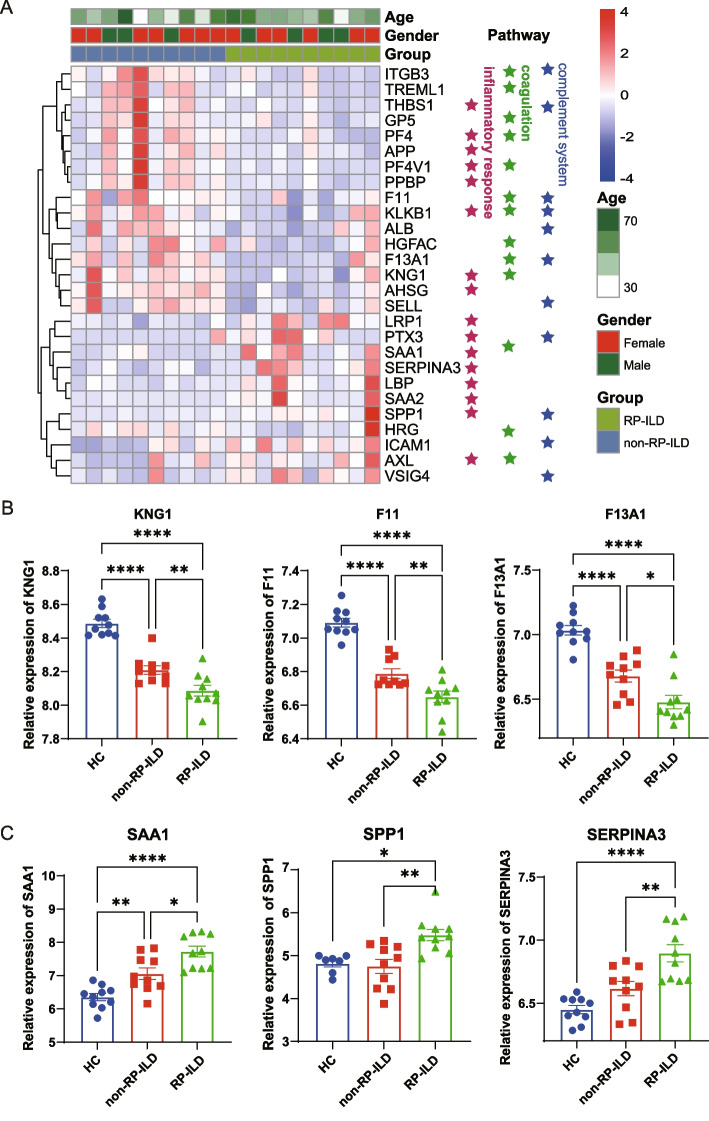
Protein expression profile of MDA5^+^ DM with RP-ILD samples. **A** Heatmap presented the proteins related to coagulation, complement system, and inflammatory response. **B**, **C** Bar plots showed the representative proteins related to the pathways. *P* values were calculated by the analysis of variance (ANOVA) test. *p < 0.05, **p < 0.01, ***p < 0.001, ****p < 0.0001

The heatmap exhibits the relevant proteins that are enriched in the above three pathways (Fig. 3A). A total of 20 proteins were enriched in the complement and coagulation pathway, including KNG1, F11, and F13A1 (Fig. 3B). We also detected significant alterations in 16 acute phase proteins and acute inflammatory response proteins in the plasma of the RP-ILD patients, including SAA1, SPP1, and SERPINA3.

**Fig. 4:**
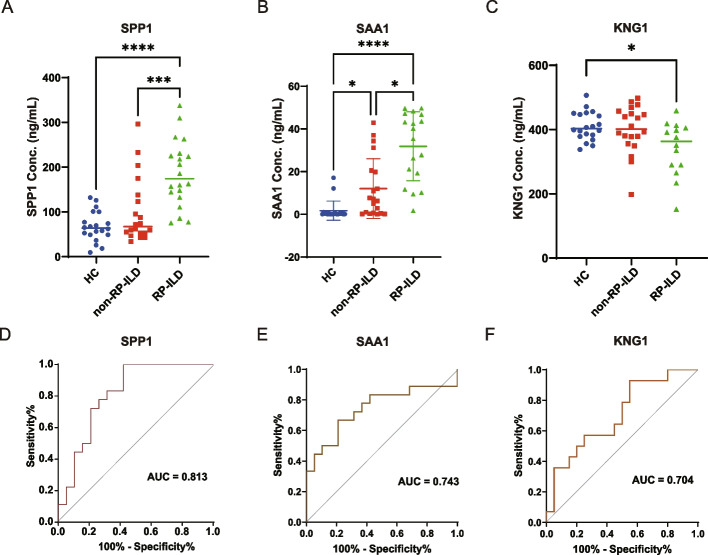
Plasma concentration of SPP1, SAA1, and KNG1 in MDA5^+^ DM RP-ILD.** A–C** The concentrations of SPP1, SAA1, and KNG1 in the plasma from an independent cohort were determined by ELISA. *P* values were calculated by the ANOVA test. *p < 0.05, **p < 0.01, ***p < 0.001, ****p < 0.0001. **D–F** ROC curve showed the AUC of the 3 verified proteins, respectively

We selected 6 candidates, including SPP1, SAA1, KNG1, F11, F13A1, and VSIG4 for validation in an independent cohort comprised of 20 RP-ILD patients, 20 non-RP patients, and 20 HC using the ELISA test. The results indicated that the concentrations of SPP1, SAA1, and KNG1 were significantly elevated in RP-ILD patients than those in non-RP-ILD patients and HC (Fig. 4A–C). However, the concentrations of VSIG4, F11, and F13A1 among the three groups of patients showed no significant difference (data not shown).

Furthermore, we performed ROC analysis to compare the sensitivity and specificity of the aforementioned protein for RP-ILD. Comparing RP-ILD and non-RP-ILD patients, the AUC for SPP1, SAA1, and KNG1 were 0.813 (*p* = 0.001), 0.743 (*p* = 0.012), and 0.704 (*p* = 0.046), respectively (Fig. 4D–F). Among these potential biomarkers, SPP1 showed the best AUC value for predicting RP-ILD. When these potential biomarkers were combined in a panel of protein expression, the AUC value was 0.938 (*p* = 0.002) in RP-ILD patients versus non-RP-ILD patients.

High levels of plasma SPP1 are observed in many different chronic and acute diseases [22–24]. To eliminate the influence of confounding factors such as arthritis, diabetes, renal disease, and cardiovascular disease on SPP1 expression, a GLM model was used. The adjusted *p*-values were found to be 0.0059 and 0.037 in the mass spectrometry and ELISA samples, respectively (data not shown).

### SPP1 was increased in high-risk subgroups for RP-ILD

**Fig. 5:**
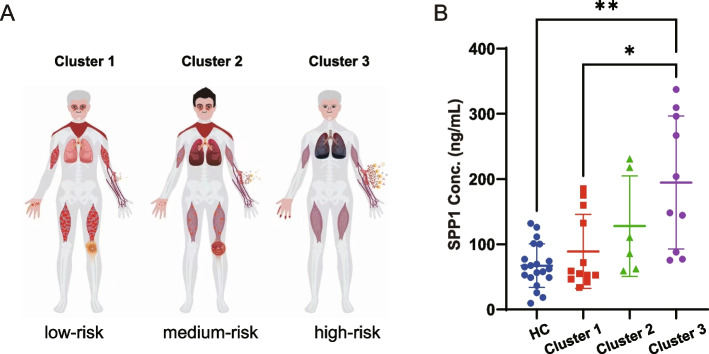
Plasma concentration of SPP1 in relation to disease subtypes. **A** The cartoon shows the three subgroups in MDA5^+^ DM patients with mild (cluster 1), moderate (cluster 2), and high RP-ILD risk (cluster 3) that were identified by our previous study. **B** Plasma concentration of SPP1 in different MDA5^+^ DM subtypes

We previously identified three subgroups in MDA5^+^ DM patients with mild (cluster 1), moderate (cluster 2), and high RP-ILD risk (cluster 3) (Fig. 5A) [2]. MDA5^+^ DM patients were then divided into 3 clusters based on our previous decision tree model. Consistently, cluster 3 had a high inflammatory status that related to high RP-ILD and mortality risk in the current study. We compared the plasma SPP1 concentration in different subgroups of patients. SPP1 exhibited an increasing trend from HC, cluster 1 to cluster 3. Cluster 3 exhibited 1.5, 1.8, and 2.9-fold higher levels of SPP1 compared to cluster 2 (*p* = 0.24), cluster 1 (*p* = 0.02), and the HC group (*p* = 0.0001), respectively (Fig. 5B). Our data suggested that SPP1 was particularly increased in high-risk RP-ILD subgroups.

## Discussion

RP-ILD is a life-threatening complication of MDA5^+^ DM, and there is currently a lack of reliable biomarkers to predict RP-ILD. Using LC–MS/MS analysis, we analyzed the protein profile in the plasma of MDA5^+^ DM and made two major novel findings. First, we identified 79 proteins that changed in the plasma of RP-ILD patients as compared with non-RP-ILD patients. These molecular changes mainly involve acute phase proteins, complement, and coagulation activation pathways, suggesting a potential role in the progression of RP-ILD. Second, our data supports plasma SPP1 as a potential biomarker for RP-ILD prediction. Taken together, our findings offer fresh insights for further elucidating the pathogenesis in MDA5^+^ DM with RP-ILD and provide valuable clues for developing promising therapeutic and prognostic biomarkers.

Our proteomic data showed a substantial activation in the biological process of inflammatory response in RP-ILD patients. MDA5^+^ DM patients tend to have high sera inflammation status, with markedly increased serum ferritin and high type I interferon [25]. These factors are associated with the severity and poor prognosis. We previously reported that MDA5^+^ DM patients with high-RP-ILD risk were characterized by elevated CRP, lactate dehydrogenase (LDH), and ferritin at baseline compared with low or medium RP-ILD risk patients, supporting our current findings that a persistent and severe inflammatory response promotes lung injury.

We revealed a variety of acute phase proteins (APPs) elevated in RP-ILD patients, including SSA1, SAA2, SERPINA3, and lipopolysaccharide-binding protein (LBP). This observation is consistent with other studies indicating that high CRP levels are associated with RP-ILD [2, 26, 27]. APP levels rise rapidly in response to inflammation or tissue damage. Proteins such as CRP and SAA1 can activate the complement system and promote the production of cytokines and chemokines [28, 29], contributing to the “cytokine storm” and acute lung injury. Our data suggest that active control of inflammation is vital for halting RP-ILD progression in MDA5^+^ DM.

The complement system plays a critical role in innate and adaptive immune responses, but its excessive activation may result in tissue injury. The role of the complement system has been well-defined in lung disease derived from coronavirus infection including COVID-19, severe acute respiratory syndrome (SARS), and Middle East respiratory syndrome (MERS) [30–32]. Excessive complement activation could induce endothelial cell injury, blood clotting, and systemic microangiopathy, eventually, resulting in multi-systemic organ failure in patients with COVID-19 [33]. Complement activation products, such as C3a and C5a, can recruit inflammatory cells to the lungs and promote the release of pro-inflammatory cytokines, contributing to lung inflammation and injury [34]. Complement inhibitors are currently not used in MDA5^+^ DM. Therefore, further exploration of the pathophysiologic importance of complement in RP-ILD is needed and could suggest a novel specific intervention for RP-ILD.

Lung is the primary site of terminal platelet production and accounts for approximately 50% of total platelet production [35]. Platelets may in turn contribute to lung injury [36, 37]. Five proteins (GP5, PF4, PF4V1, TREML1, ITGB3) that were associated with platelet aggregation and adhesion were found to be downregulated in our data. Moreover, the suppressed platelet degranulation was observed in the plasma of MDA5^+^ DM patients with RP-ILD. In addition to regulating thrombosis, activated platelets could directly interact with immune cells, thereby promoting an inflammatory phenotype [38]. In acute respiratory distress syndrome (ARDS) animal model or clinical data from the study of bronchoalveolar lavage fluid in ARDS patients, platelets are proven to contribute to the development of acute lung injury [39]. Data from animal models [40, 41] and human studies [42, 43] have suggested antiplatelet therapies with aspirin could reduce incidence and mortality in ARDS. Collectively, our evidence suggests that the inhibition of complement activation and inflammatory responses, as well as antiplatelet therapies, might be helpful in the treatment of MDA5^+^ DM patients with RP-ILD.

With the SARS-CoV-2 pandemic outbreak worldwide, increasing evidence showed the striking similarities between COVID-19 and MDA5^+^ DM with RP-ILD, including chest computed tomography feature, high serum cytokine levels, severe acute respiratory symptoms, and high mortality, suggesting these two conditions share common pathophysiological mechanisms. Interestingly, recent studies have revealed that complement activation, complement-induced inflammatory response, and blood clotting were particularly evident in severe COVID-19 patients in response to viral infection [33, 44, 45]. How certain MDA5^+^ DM patients develop RP-ILD remains largely unclear. Viral infection has long been considered a suspected trigger of disease progression in MDA5^+^ DM patients. Our data imply that COVID-19 and MDA5^+^ DM may share a common underlying molecular mechanism for lung injury.

We validated 6 proteins and found SAA1, SPP1, and KNG1 are associated with the risk of RP-ILD. Among them, SPP1 showed the best predictive value. SPP1, also known as osteopontin, is involved in a variety of physiological and pathological processes including wound healing, bone turnover, tumorigenesis, inflammation, ischemia, and immune responses [46, 47]. SPP1 is expressed by a variety of inflammatory cells in culture, including T cells, macrophages, and NK cells [48–50]. During inflammation, SPP1 could induce cell adhesion and migration, mediate proinflammatory lymphocytes activation and cytokine production, and inhibit the apoptosis of inflammatory cells [51, 52]. Single-cell transcriptome analysis found that SPP1 was significantly higher in bronchoalveolar lavage in severe cases of COVID-19 compared to control and mild cases [53]. Levels of SPP1 were also found to correlate with highly aggressive lung adenocarcinoma [54].

In addition to participating in inflammatory processes, increasing evidence suggests that the upregulation of SPP1 is associated with fibrosis. Anna et al. conducted a study where they performed single-cell RNA sequencing on 11 explanted lungs from patients with systemic sclerosis-associated ILD, and they identified the presence of SPP1^+^ profibrotic macrophages [55]. Furthermore, studies have demonstrated that the downregulation of SPP1 effectively reduces pulmonary fibrosis in a bleomycin-induced pulmonary fibrosis mouse model [56]. Given that both inflammation and fibrosis are crucial pathological processes in the development of ILD, it is not surprising that SPP1 is linked to ILD risk in MDA5^+^ DM patients. The exact mechanism requires further validation through experiments.

Previous studies have found that SPP1/osteopontin was upregulated in MDA5^+^ DM [57, 58]. Based on the risk stratification, we found that SPP1 was specifically increased in the high-risk RP-ILD subgroup. The typical clinical features of the high-risk RP-ILD subgroup are the elevated levels of sera inflammation markers (ESR, CRP, ALT, AST, and LDH), as compared with the low- or medium-risk subgroup. These results demonstrate that SPP1 could serve as a novel biomarker for monitoring RP-ILD progression, and has the potential for developing new therapies for RP-ILD treatment.

In our study, we also observed SAA1 were upregulated in MDA5^+^DM-RPILD. As an acute-phase protein, SAA1 plays a role in regulating inflammation and immunity. Previous studies have reported its upregulation in fibrotic sarcoidosis [59] and COVID-19-related ARDS [60]. However, further research is needed to elucidate the specific mechanisms by which SAA1 contributes to interstitial lung lesions.

Several limitations remain in this study. First, the sample size was limited, and the role of identified biomarkers or pathways should be validated in a larger sample prospective cohort. Second, dynamic observations will be necessary to evaluate the effect of the treatments on the expression of the differential proteins. Third, the molecular functions of the differential proteins should be examined in further study to understand their role in the pathogenesis of MDA5^+^ DM.

## Conclusions

Our study provides novel insights into the pathogenesis of RP-ILD development in MDA5^+^ DM and highlights the significance of plasma SPP1 as both a novel prognostic biomarker and a therapeutic target for MDA5^+^ DM.

### Supplementary Information

**Additional file 1:** **Table S1.** Differentially expressed proteins of MDA5^+^ DM versus healthy control.

**Additional file 2:** **Table S2.** Differentially expressed proteins of MDA5^+^ DM with RP-ILD versus MDA5^+^ DM without RP-ILD.

## Data Availability

The data from this article will be shared upon reasonable request to the corresponding author.

## Abbreviations

APPs: Acute phase proteins
ARDS: Acute respiratory distress syndrome
AUC: Receiver operating characteristic curve
GLM: Generalized linear model
CRP: C-reactive protein
DEPs: Differentially expressed proteins
DM: Dermatomyositis
ELISA: Enzyme-linked immunosorbent assay
ESR: Erythrocyte sedimentation rate
F11: Coagulation Factor XI
F13A1: Coagulation Factor XIII A Chain
HC: Healthy controls
IIM: Idiopathic inflammatory myopathies
ILD: Interstitial lung disease
KL-6: Krebs von den Lungen-6
KNG1: Kininogen 1
LBP: Lipopolysaccharide-binding protein
LC–MS/MS: Liquid chromatography-tandem mass spectrometry
LDH: Lactate dehydrogenase
MDA5 +: Anti-melanoma differentiation-associated gene five antibody positive
MERS: Middle East respiratory syndrome
PCA: Principal component analysis
ROC: Receiver operating characteristic
RP-ILD: Rapidly progressive interstitial lung disease
SAA1: Serum amyloid A1
SARS: Severe acute respiratory syndrome
SPP1: Secreted phosphoprotein 1
STEM: Short Time-series Expression Miner
VSIG4: V-Set And Immunoglobulin Domain Containing 4
